# Intracranial hypertension and cerebellar symptoms due to Lhermitte-Duclos disease

**Published:** 2015-04-04

**Authors:** Farhad Assarzadegan, Atoosa Gharib, Shirin Behbahani, Meysam Ebrahimi-Abyaneh

**Affiliations:** 1 Department of Neurology, Imam Hossein Hospital, Shahid Beheshti University of Medical Sciences, Tehran, Iran; 2 Department of Pathology, Imam Hossein Hospital, Shahid Beheshti University of Medical Sciences, Tehran, Iran; 3 Department of Neurosurgery, Imam Hossein Hospital, Shahid Beheshti University of Medical Science, Tehran, Iran

**Keywords:** Lhermitte-Duclos Disease, Dysplastic Gangliocytoma cerebellum, Magnetic Resonance Imaging

Lhermitte-Duclos disease (LDD) is a rare pathologic condition, which was first described in 1920 by Giorgianni et al.^1^ This is a slowly growing tumor of the cerebellum, composed of granule Purkinje and glia cells. It evolves in a disorganized fashion and the cerebellum loses its normal architecture with no clear plane from normally structured cerebellar tissue.^2^^-^^5^ We report a case of a young woman with hydrocephalus and cerebellar symptoms caused by a rare occurrence of dysplastic Gangliocytoma arising within the left cerebellar hemisphere and vermis.

A 19-year-old woman with uneventful medical history was admitted in March 2012 with a 6 months history of vertigo and the posterior headache and 2 months history of nausea, vomiting, and visual disturbance. On admission, the neurologic examination revealed edema in fundoscopy and an unsteady tandem gait with a tendency to fall to the left side. Other neurologic exams were normal. Computed tomography (CT) scan showed a hypodense area in the left cerebellar hemisphere and vermis with no calcification, reduced fourth ventricle, and obstructive hydrocephalus. Magnetic resonance imaging (MRI) with and without contrast revealed an irregular lesion, hypo signal on T1-weighted image and high signal on T2-weighted image with no enhancement (Figure 1). The patient was submitted to surgery through a left sub occipital craniotomy and a gross total resection of the tumor and subtotal left side of the cerebellum. Upon opening the dura matter a very large, wide, gray-colored cerebellar folia were visualized expanding throughout the left cerebellum and vermis. The patient recovered uneventfully with resolution of the neurologic symptoms. She was discharged from hospital 7 days after the surgery. The surgical piece showed enlargement and hypertrophy of the cerebellar cortex and folia. On histologic examination, the internal granular cell layer completely replaced the Purkinje cell. Normal Purkinje cell were absent (Figure 2). The final diagnosis was of dysplastic gangliocytoma of the cerebellum, World Health Organization (WHO) Grade 1.

LDD is a rare condition, usually affecting patients aged 30-50 years. There is no sex preference.^4^ Around 220 LDD cases, have been reported in the literature.^1^^,^^5^ This is a slowly evolving lesion that forms a mass. It is composed of granule, Purkinje and glia cell. LDD was found associated with phakomatosis, Cowden syndrome, systemic hamartomas and malignant neoplastic lesions of the breast, thyroid and genito urinary tract.^2^^,^^3^ Imaging plays an important role. Hypo attenuated on unenhanced CT scan. No appreciable enhancement is seen on contrast-enhanced CT image.^1^

**Figure 1:**
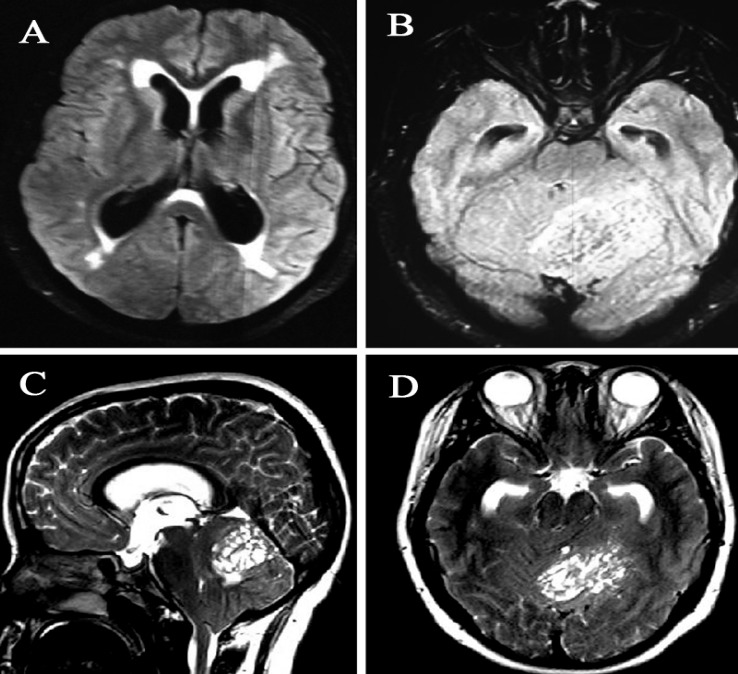
Axial T2-fluid-attenuated inversion recovery magnetic resonance imaging showing hypersignal mass in the left cerebellar hemisphere (A) and hydrocephalus due to compression of forth ventricle (B). T2-sequence MRI sagittal and axial sections showing the characteristic “tiger stripe” appearance of this hamartomatous tumor in the left cerebellar hemisphere (C and D)

**Figure 2:**
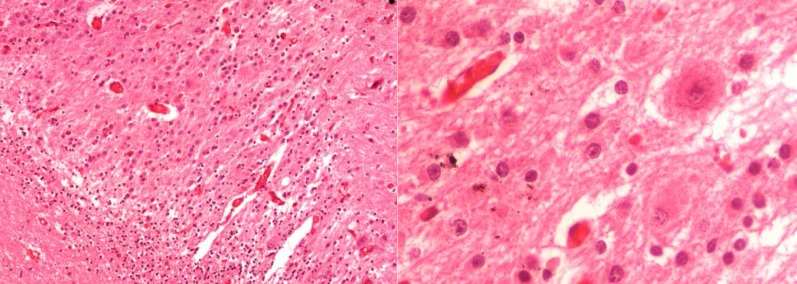
In histologic examination the internal granular cell layer was completely replaced Purkinj’s cell

The appearance on MRI imaging is highly characteristic, showing tiger stripe appearance. LDD should be excised if symptomatic.^2^

In this case report, we presented a patient with dysplastic gangliocytoma of the cerebellum both as hemisphere and vermis with symptoms of cerebellum and raised intracranial pressure. The diagnosis of this rare entity should be considered in any young, and middle age adult presenting with signs of intracranial hypertension and cerebellar combined with characteristic radiologic features; however MRI cannot replace the histopathologic diagnosis. Surgery appears to be the only efficient treatment if symptomatic. Long-term follow up is advisable in order to reduce the probability of occasional symptomatic recurrence and to identify the possible sign of Cowden syndrome, which carries a risk of developing malignancy.

## Notes:

**How to cite this article: **Assarzadegan F, Gharib A, Behbahani Sh, Ebrahimi-Abyaneh M. Intracranial hypertension and cerebellar symptoms due to Lhermitte-Duclos disease. Iran J Neurol 2015; 14(2): 113-5.
